# Iron metabolism and hematological abnormalities in adult patients affected with mucopolysaccharidoses

**DOI:** 10.1016/j.ymgmr.2025.101243

**Published:** 2025-07-16

**Authors:** Gabija Kaciulyte, Goknur Yorulmaz, Reena Sharma, Simon A. Jones, Robert Wynn, Heather Church, Karen Tylee, Muzaffer Bilgin, Ana Jovanovic, Peter Woolfson, Karolina M. Stepien

**Affiliations:** a Faculty of Biology, Medicine, and Health, The University of Manchester, UK; b Faculty of Medicine, Eskisehir Osmangazi University, Eskisehir, Turkey; c Salford Royal Hospital, Northern Care Alliance NHS Foundation Trust, UK; d Willink Biochemical Genetics, St Mary's Hospital, Manchester Foundation Trust, Manchester, UK; e Department of Pediatrics Hematology, Manchester University NHS Foundation Trust, Manchester, UK; f Cardiovascular Sciences, University of Manchester, Manchester, UK

**Keywords:** Iron metabolism, Mucopolysaccharidosis, Iron deficiency, Hematopoietic stem cell transplantation, Enzyme replacement therapy, Cardiac surgery, Inflammation

## Abstract

Mucopolysaccharidoses are a heterogeneous group of rare lysosomal storage disorders (LSDs) caused by genetic mutations resulting in the deficiency of lysosomal enzymes responsible for the degradation of glycosaminoglycans (GAGs). The potential association of hematological abnormalities and clinical manifestations in MPS disorder highlights the importance of exploring the role of regular iron studies and hematological tests in at risk MPS patients as undiagnosed anemia has been shown to worsen clinical outcomes. In this study, therefore, we aimed to describe the hematological abnormalities and iron studies in adult patients with MPS disorders in the context of their therapies. Ninety-seven patients with MPS types I, II, III, IV and VI were included in the study (46 % females). The overall prevalence of iron deficiency anemia is 38 % in adult MPS cohort and affects 63 % of MPS III and 50 % of MPS I patients. It is more common in females with MPS I and IVA than males, post cardiac valve surgery and in patients with gastrointestinal dysfunction. There was high variability in Hb, mean corpuscular volume, serum iron and transferrin saturation among all the MPS subgroups, with results being higher in the HSCT and ERT group as compared to treatment naïve patients. The urine GAGs/creatine ratio negatively correlated with several critical parameters, including iron saturation (−0.07), serum iron (−0.06), Hb (−0.16), and hematocrit (−0.08). It was more pronounced among MPS III patients. Pancytopenia in treatment naive MPS III is associated with 7 to 20 fold increase of urine GAGs excretion. It is important to take into consideration hematological and iron study abnormalities in the management of MPS patients, as special approaches may be required for both intestinal health and anemia treatment.

## 1 Introduction

Mucopolysaccharidoses (MPS; I, II, III, IV, VI, VII) are a heterogenous group of rare lysosomal storage disorders (LSDs) caused by genetic mutations resulting in the deficiency of lysosomal enzymes responsible for the degradation of glycosaminoglycans (GAGs) i.e. keratan, heparan, chondroitin and dermatan sulphate [1]. The systemic accumulation of GAGs in blood, cerebrospinal fluid and tissues affects several internal organs and major systems, which manifests as progressive cognitive deterioration, joint stiffness, cardiac valve disease, gastrointestinal and respiratory dysfunction [3]. The severity of MPS is variable with lethal forms in utero and attenuated forms diagnosed in adults [2]. The natural history of the MPS disorders is not fully understood.

Bone marrow involvement and anemia, leukopenia, thrombocytopenia, bicytopenia / pancytopenia and associated complications can be seen in all patients with LSDs i.e. Gaucher [4], Niemann Pick C or B [5]. While thrombocytopenia is more common in LSDs, anemia and leukopenia are less frequent. If anemia is not a component of pancytopenia, it can be easily overlooked. If bicytopenia / pancytopenia is present, caution should be exercised in terms of hypersplenism that may accompany the involvement of the bone marrow. The evidence on bone marrow infiltration and its findings in MPS disorders is limited.

Despite the very limited data on anemia, there were attempts to evaluate leucocyte and platelet function in MPS disorders. The changes in leucocytes in MPS II and VI with the enzyme replacement therapy (ERT) have been described before [6]. It was suggested that the type of substrate accumulated and/or enzyme deficiency in the lysosome may have a particular effect on the normal cellular composition of the immune system. A study on a pediatric MPS group has demonstrated that mean platelet volume (MPV) as an indicator of platelet function and activation, has remained low during infection or chronic inflammation [7]. Uz et al. [8] described idiopathic thrombocytopenic purpura in a case diagnosed with Hunter syndrome (MPS II) (Uz, 2012).

Given the chronic inflammation and elevation of interleukin 6, as a possible underlying pathophysiology in MPS disorders [9], bone marrow involvement, gastroenteritis, airway obstruction and malnutrition are therefore possible contributors of hematological abnormalities in MPS patients [10]. The evidence comes from clinical studies describing patients with chronic illnesses. In contrary, the analysis of a data from a clinically stable pediatric population showed no hematological l abnormalities, apart from one case of cyclical neutropenia [11].

However, the literature regarding the prevalence of hematological abnormalities in the adult MPS cohort is sparse.

The potential association of hematological abnormalities and clinical manifestations in MPS disorder highlights the importance of exploring the role of regular iron studies and hematological tests in at risk MPS patients as undiagnosed anemia has been shown to worsen clinical outcomes [12]. In this study, therefore, we aimed to describe the differences in iron deficiency anemia (IDA) and hematological abnormalities in adult patients with various MPS disorders in the context of their therapies.

## 2 Materials and methods

### 2.1 Study design

It was a service evaluation study registered within the Research and Innovation department, Salford Royal Hospital, Northern Care Alliance NHS Foundation Trust, United Kingdom (22HIP27).

### 2.2 Patients

Adult patients (above the age of 16) with MPS type I, II, III, IV, VI attending Adult Metabolic Clinics were included in the study. Age, gender, MPS type, and treatment type; enzyme replacement therapy (ERT) or hematopoietic stem cell transplantation (HSCT) were extracted. Patients were diagnosed with MPS in childhood and transitioned to adult services for a long-term follow-up. Some presented with attenuated forms in adulthood.

### 2.3 Biochemistry tests

The results of serum hemoglobin (Hb), serum iron, transferrin saturation, ferritin, serum B12, serum transferrin, serum folate, mean corpuscular volume (MCV) and urine GAGs were obtained from the database. All results are requested as part of routine clinical practice every 6–12 months. Blood tests were collected during clinic visits and several days after the surgical procedures. As inflammatory markers (CRP, ESR) is not measured routinely, they were not included in this analysis. White cell count was measured as part of the full blood count.

### 2.4 Statistical analysis

Descriptive statistics mean (SD) and median (range) were used to describe patients' demographic and clinical characteristics for continuous variables. Percentages were calculated for categorical variables. The results were presented as means ± SD. The R Project for Statistical Computing program was used in statistical analysis. A *p*-value ≤0.05 was considered statistically significant.

Compliance with normal distribution was tested with the Shapiro Wilk test. Spearman correlation test was used to determine the direction and magnitude of the relationship between variables that were not normally distributed.

All analyses were performed with parametric evaluations.

### 2.5 Definitions

Iron deficiency anemia occurs when human body lacks sufficient iron, leading to a reduced ability to produce red blood cells, which carry oxygen to the body's tissues. World Health Organization (WHO) age-specific cut-offs for Hb are 130 g/L for adult males and 120 g/L for adult females [13], which are indicators of anemia. Patients with anemia are further categorized in microcytic (MCV <80 fL), normocytic (80–100 fL) and macrocytic (>100 fL).

## 3 Results

### 3.1 Basic characteristics

Ninety-seven patients with five MPS types (I, II, III, IV and VI) were included in the study; 45 were females (46 %) and 52 were males (54 %). Since MPS II is inherited in an X-linked recessive pattern, all cases (12) were male. Hematopoietic stem cell transplantation (HSCT) was performed in 26 patients (26.8 %), including 25 patients with MPS I and one patient with MPS VI. The median length of follow up after the HSCT was 28 years. Forty-three patients (44 %) were receiving enzyme replacement therapy (ERT). The median length of ERT treatment was 18 years. A total of 29 patients (30 %), 10 of whom had MPS III, were treatment naive. Majority of patients were able to eat independently. In a small proportion (*n* = 2) gastrostomy feeding was applied.

In this study, heart valve replacement (metallic valve) was performed in six patients with MPS I. Their urinary GAG concentration was higher in the heart valve replacement group (*p* < 0.026), with serum iron (p < 0,028), Hb (*p* < 0.009), hematocrit(*p* < 0.002) and MCV (*p* < 0.032) levels being statistically significantly low.

Blood samples were taken at baseline before iron replacement was recommended. Some of them take non-steroidal inflammatory drugs for joint pain, some take steroids as a premedication and none of them was taking antiplatelets agents.

Blood parameters and urine GAGs of the patients are summarized in Table 1.

**Table 1:** Mean (±SD) of hemoglobin (Hb), mean corpuscular volume (MCV), serum iron, iron saturation, urine GAGs respective of MPS type and therapy. ERT-enzyme replacement therapy; HSCT-hematopoietic stem cell transplantation; none- no therapy.

MPS type	Treatment	Age	Hb (130–180 g/L)	MCV (84–105 fL)	Serum iron (9–30 μmol/L)	Transferrin Saturation (15–45 %)	Urine GAGs (mg/mmol)
MPS I	None (n:2)	58.0 ± 16.9	112.0 ± 5.7	85.7 ± 1.2	9.0 ± 0.1	14.30 ± 6.08	5.70 ± 1.41
	ERT (n:15)	29.4 ± 11.7	131.5 ± 20.6	85.4 ± 7.3	10.7 ± 6.7	16.47 ± 11.51	5.35 ± 3.28
	HSCT (n:25)	27.6 ± 7.4	126.1 ± 30.4	91.9 ± 11.2	12.3 ± 5.7	17.77 ± 9.28	6.41 ± 3.29
MPS II	None (n:3)	42.3 ± 15.7	150.33 ± 14.64	93.07 ± 4.44	16.13 ± 6.28	24.67 ± 16.74	23.13 ± 18.21
	ERT (n:9)	28.4 ± 6.0	144.78 ± 17.17	86.69 ± 6.97	15.83 ± 7.05	25.00 ± 11.27	5.76 ± 1.88
MPS III	None (n:10)	37.2 ± 15.4	125.80 ± 14.15	82.80 ± 27.95	12.52 ± 5.04	20.00 ± 10.26	59.12 ± 65.48
MPS IV	None (n:10)	32.1 ± 13.5	136.70 ± 19.46	79.51 ± 28.19	12.50 ± 4.97	17.30 ± 8.38	7.97 ± 2.76
	ERT (n:13)	27.1 ± 7.8	138.08 ± 14.09	86.92 ± 5.50	17.15 ± 7.63	24.46 ± 11.30	6.62 ± 2.35
	None (n:3)	36.7 ± 8.9	146.33 ± 8.96	87.57 ± 3.27	10.1 ± 6.9	16.33 ± 10.07	11.30 ± 9.87
MPS VI	ERT (n:6)	27.0 ± 8.4	136.33 ± 13.74	85.43 ± 5.1	12.3 ± 8.0	20.00 ± 20.00	5.80 ± 1.65
	HSCT (n:1)	35.0	145.00 ± 0	93.90 ± 0	14.40 ± 0	29.00 ± 0	4.60 ± 0

#### 3.1.1 Hemoglobin

Female patients with MPS I have statistically significantly lower Hb as compared to males (Table 2). Those who receive ERT have higher average Hb levels compared to those with no treatment or those who underwent HSCT. However, there is a substantial variability in Hb levels among patients who underwent HSCT (Table 1). Treatment naïve MPS II patients have slightly higher average Hb levels compared to those receiving ERT. The differences are relatively small, and both groups have high Hb levels. Among the MPS III group, the average Hb level was 125.80 ± 14.15 g/dL. In MPS IV, Hb levels are similar between untreated patients and those receiving ERT, with ERT patients having slightly higher levels. Females with MPS IVA have significantly lower Hb as compared to males (Table 2).

**Table 2:** Differences between male and female patients with MPS I, III, IVA and VI. MPS II was excluded from the subanalysis (only males).

	Serum iron (median values)			Iron saturation (median values)			Hb (median values)			MCV (median values)		
	M	F	*p* value	M		p value	M	F	P value	M	F	P value
MPS I (21 M/22F)	14.75	13	**0.0006**	8.88	22.5	**0.0012**	141	113	**0.0004**	91.5	85.3	**0.006**
MPS III (5 M/5F)	10.4	16.4	0.204	14.6	24.8	0.185	127	123	0.6	90	79	0.5
MPS IVA 9 M/14F)	19.1	27.7	**0.02**	12.5	17.2	**0.015**	150	129	**0.001**	86	79.6	0.4
MPS VI (5 M/5F)	12.8	20	0.66	10.8	19.6	0.97	89	84	0.13	89	84	0.13

Among patients with MPS VI, untreated individuals have the highest average Hb levels, followed closely by those who underwent HSCT. Patients receiving ERT have slightly lower Hb levels compared to the untreated group. Overall, Hb levels vary across different MPS types and treatments, with some treatments showing higher levels compared to no treatment, while others are relatively similar.

#### 3.1.2 MCV

Patients with MPS I, who underwent HSCT have higher average MCV levels compared to those with no treatment or those receiving ERT. The MCV levels for untreated and ERT-treated patients are very similar (Table 1). MCV was statistically significantly lower in females as compared to males with MPS I (Table 2).

Treatment naïve patients with MPS II have higher average MCV levels compared to those receiving ERT. There is a notable difference between the two groups.

Treatment naïve patients MPS III had a mean MCV of 82.80 ± 27.95 fL. Three MPS III adults who had high MCV > 100 fL had pancytopenia like picture, which corresponded with enlarged spleen (16, 16 and 14 cm) and very high urine GAGs/creatinine ratios (220, 102 and 72).

Patients with MPS IVA who receive ERT have higher average MCV levels compared to the treatment naïve ones. The untreated group shows a high degree of variability. One adult patient with MPS VI who underwent HSCT in childhood, had the highest MCV level. Treatment naïve and ERT-treated MPS VI patients had similar MCV levels.

Overall, MCV levels vary across different MPS types and respective of the therapeutic modality. HSCT generally results in higher MCV levels in the available data. There was positive correlations between MCV and serum B12 and serum folate (0.27) (Fig. 2).

#### 3.1.3 Serum iron/ iron saturation

Patients with MPS I post HSCT have the highest average iron saturation levels, followed by those receiving ERT and then treatment naïve group. There is considerable variability in iron saturation levels among patients on ERT. There was a statistically significant difference in serum iron and iron saturation among females and males with MPS I (Table 2).

In patients with MPS II, the iron saturation levels were very similar between treatment naive patients and those receiving ERT. Both groups have high average iron saturation levels with considerable variability.

Among treatment naïve MPS III patients, an average iron saturation level was 20.00 ± 10.26 %.

Patients with MPS IVA who received ERT had higher average iron saturation levels compared to the treatment naïve group. There was a statistically significant difference in serum iron and iron saturation among females and males with MPS IVA (Table 2).

One MPS VI patient post HSCT had the highest iron saturation level. The ERT-treated group has slightly higher average iron saturation levels compared to the treatment naïve group, but both groups show variability.

Overall, iron saturation levels vary across different MPS types and treatments, with higher levels in the treatment group. ERT treatment generally improves iron saturation levels compared to the treatment naïve group.

#### 3.1.4 Ferritin

Median serum ferritin was 48.5 μg/L (range 6–133) for a subgroup of 25 patients. High values of 400–550 μg/L were reported in hospitalized patients and excluded from this sub-analysis.

#### 3.1.5 Serum B12 and folate

Median serum vitamin B12 was 494 ng/l (range 88–2000; NR 211-911 ng/L) and median serum folate was 7.98 μg/l (range 1.7–24; NR > 4μg/L) for the total cohort of MPS patients. There were no significant differences among the MPS types or among two genders (calculated for MPS I, III, IVA and VI).

### 3.2 Serum iron in MPS patients

**Fig. 1:**
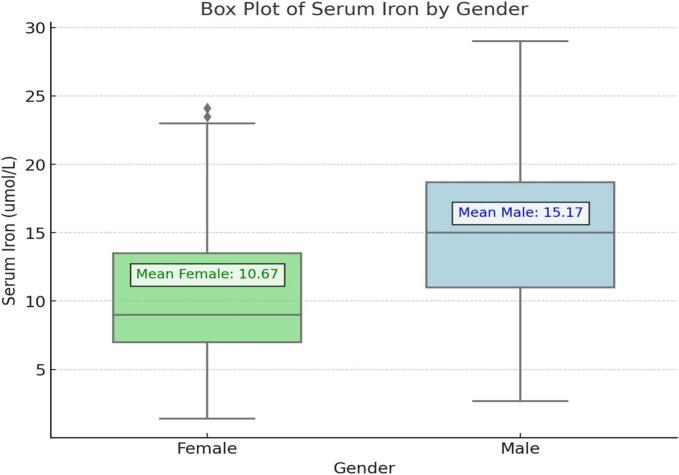
Serum iron in females and males with MPS. The mean serum iron level is 10.67 μg/dL, indicated by the green text and arrow. The mean serum iron level is 15.17 μg/dL, indicated by the blue text and arrow. Female patients have a lower mean serum iron level compared to male patients. The range and IQR for males are broader than those for females, indicating more variability in serum iron levels among male patients. The median serum iron level is higher for males than for females. The variability in serum iron levels is greater among males, which is evident from the wider spread in the box plot.

Mean values  of serum iron levels were calculated according to gender in MPS groups (Fig. 1). Urine GAGs levels of the patients showed a negative correlation with iron levels (Supplementary Fig. 1).

**Fig. 2:**
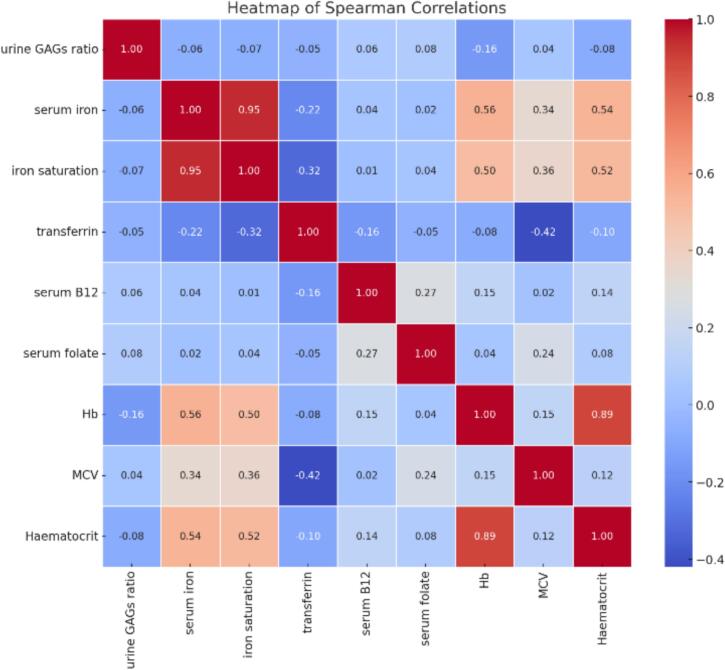
Spearman correlation heatmap of hematologic and biochemical parameters in MPS patients; The provided heatmap illustrates the Spearman correlation coefficients among various hematologic and biochemical parameters in MPS patients. Notably, the urine GAGs ratio shows a negative correlation with several critical parameters, including iron saturation (−0.07), serum iron (−0.06), hemoglobin (Hb) (−0.16), and hematocrit (−0.08), suggesting that higher urine GAGs levels are associated with lower levels of these parameters. Serum iron is strongly positively correlated with iron saturation (0.95) and moderately positively correlated with Hb (0.56) and hematocrit (0.54), indicating that higher serum iron levels are associated with higher iron saturation and better hematologic status. Transferrin, on the other hand, shows a negative correlation with serum iron (−0.22), iron saturation (−0.32), and MCV (−0.42), implying that higher transferrin levels might be associated with lower iron metrics. Positive correlations between serum B12 and serum folate (0.27), and between Hb and hematocrit (0.89), are also observed, reinforcing their expected biological relationships. This heatmap provides a comprehensive overview of the interrelationships among these parameters, highlighting the complex interactions within the biochemical and hematologic parameters in MPS cohort.

Urine GAGs levels showed a negative correlation with Hb, hematocrit, MCV, iron saturation and transferrin (Fig. 2).

**Fig. 3:**
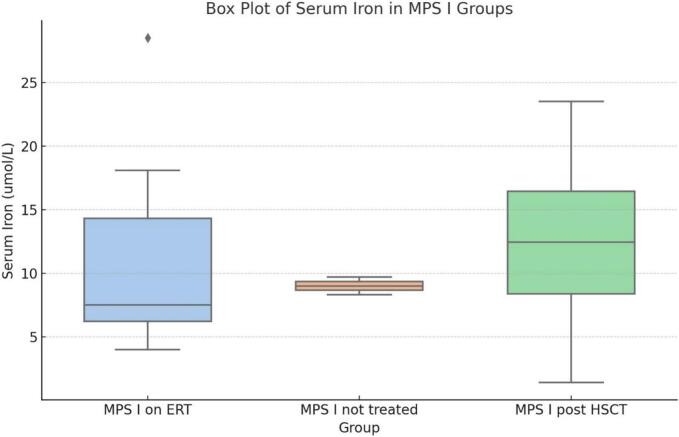
Evaluation of iron status according to treatment options in MPS I. The box plot illustrates the distribution of serum iron levels among MPS I patients across three different treatment groups: treatment naïve, on ERT, and post-HSCT. The plot shows that the untreated and HSCT groups have similar and relatively lower serum iron levels, with minimal variability. In contrast, the ERT group displays a wider range of serum iron levels, indicated by the larger interquartile range (IQR) and the presence of an outlier above the whisker. The median serum iron level in the ERT group is also higher compared to the other two groups. This variability and higher median suggest that ERT may have a more pronounced impact on serum iron levels in MPS I patients compared to treatment naïve or HSCT groups.

Among MPS I patients, serum iron was the lowest in the ERT subgroup (Fig. 3).

Heart valve replacement was performed in six of the patients followed with MPS I (4 patients post HSCT and 2 on ERT). Among patients with MPS I, urinary GAG levels were higher in heart valve replacement group (*p* < 0.026); serum iron (p < 0,028), Hb (p < 0,009), hematocrit (p 〈0,002) and MCV (p 〈0,032) levels were low (data not presented).

All patients with diagnosed iron deficiency were treated with enteral iron replacement. Four women required intravenous iron infusion: two patients with MPS IH post HSCT, one with MPS I on ERT and one patient with MPS IVA. Detailed hematology and gastroenterology investigations excluded celiac diseases and inflammatory bowel disease in them.

**Fig. 4:**
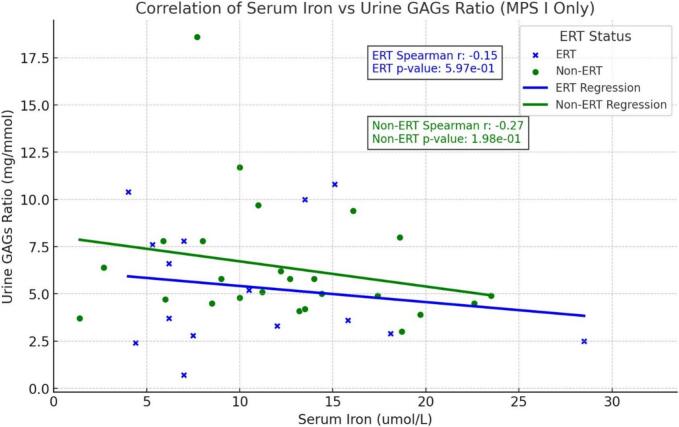
Relationship between serum iron and urine GAG in patients with MPS I on ERT and not on ERT (HSCT). Blue crosses represent ERT patients, while green crosses represent non-ERT (HSCT) patients. The plot includes fitted lines for both groups, indicating their respective trends. For the ERT group, the Spearman correlation coefficient is *r* = −0.15 with a *p*-value >0.05, suggesting a very weak and non-significant negative correlation. In contrast, the non-ERT group has a Spearman correlation coefficient of *r* = −0.27 with a p-value >0.05, indicating a weak and non-significant negative correlation. These results suggest that there is no significant relationship between serum iron levels and urine GAGs ratio in either group, though the trends slightly differ between ERT and non-ERT patients.

The relationship between serum iron and urine GAG  levels in the ERT and non-ERT groups in patients with MPS I is summarized in Fig. 4.

## 4 Discussion

This cross-sectional analysis presents abnormalities in iron studies in a large cohort of adult patients with various MPS disorders. Although iron deficiency anemia is relatively common in the general population with 2.9 % population in the North America [14] and up to 3 % for males and 8 % for females in the UK [15] and up to 6 % in Western Europe [16]. It is the commonest causes of anemia in the general population.

This study has shown that the underlying metabolic condition imposes additional contributing factors to abnormalities in iron metabolism in MPS disorders, which results in the overall iron deficiency anemia of 38 % for the adult MPS cohort. 63 % of MPS III patients had iron deficiency anemia, followed by 50 % of MPS I, 21.7 % MPS IVA, 20 % of MPS VI and 8.3 % of MPS II. There was a striking difference among females and males affected with MPS I and IVA with females being affected more with iron deficiency anemia and requiring treatment more often than males.

The study identified several of them and discusses the impact of co-morbidities, disease modifying therapies, heart valve surgery on the hematological and biochemical results.

### 4.1 Iron homeostasis in lysosomal storage diseases

Iron is essential for vital biological functions such as oxygen transport, mitochondrial energy production, DNA synthesis, and DNA repair [17]. Lysosome plays a pivotal role in iron homeostasis and lysosomal dysfunction observed in aging and lysosomal storage diseases (LSDs), can be a major factor limiting function and longevity of the human body. There is increasing evidence that iron is one of the factors likely to play a substantive role in neurodegeneration of patients with LSDs [17].

In patients presenting with iron overload, intracellular accumulation of iron has been shown to be increased in the lysosome [18]. This mechanism is thought to have a protective role [19]. The excessive iron deposits in the lysosomes can also impair lysosomal function, and lead to cellular injury through the release of hydrolytic enzymes and iron into the cytoplasm.

The iron homeostasis has been shown to be affected in Batten's disease (CLN), Gaucher disease [20], Mucolipidosis type IV [21]. The excessive accumulation of iron in the lysosome may not however reflect the serum iron concentration changes in patients with various LSDs, including MPS disorders.

### 4.2 Potential causes of iron deficiency in MPS disorders

#### 4.2.1 Gastrointestinal/nutritional

Many MPS disease manifestations are possible contributors to iron metabolism and erythropoiesis disruption such as chronic inflammation, bone marrow involvement, gastroenteritis, airway obstruction, and malnutrition. Gastrointestinal symptoms and signs such as constipation, diarrhea, food intolerance, recto-vaginal fistulas, food malabsorption, intestinal volvulus, intestinal stasis, ulcerative colitis, gastrointestinal bleeding, and pseudo-obstruction have been frequently reported in patients with LSD, including MPS disorders [22]. Some patients with MPS type III have been diagnosed with irritable bowel syndrome or Crohn's disease [22].

Untreated MPS patients, who are likely to manifest gastrointestinal symptoms, have significantly higher urine GAGs concentration compared to those receiving ERT. The provided heatmap illustrates the Spearman correlation coefficients among various hematologic and biochemical parameters in MPS patients (Fig. 2). Notably, the urine GAGs ratio shows a negative correlation with several critical parameters, including iron saturation (−0.07), serum iron (−0.06), Hb (−0.16), and hematocrit (−0.08), suggesting that higher urine GAGs levels are associated with lower levels of these parameters. Also mean values  of iron levels were calculated according to gender in MPS groups and we found female levels were low. This suggests that menstrual loss in women may make it more difficult to control iron deficiency.

### 4.3 Inflammation

In patients with inflammatory bowel disease (Crohn's disease or ulcerative colitis), an extraction of total urine GAG and chondroitin sulfate fraction was significantly higher as compared to the amount excreted in healthy subjects. It has been postulated that GAGs are involved in the disease process and therefore, urine GAGs excretion can be used as auxiliary test in the diagnosis of patients with inflammatory bowel disease [23].

In a study conducted by Oshiro et al., heparan sulfate was shown to be localized to the basolateral surfaces of the small intestine and stomach and stored in these regions. Similarly, a study evaluating the gastrointestinal tract in MPS IIIA mice showed lysosomal GAG accumulation in the lamina propria of the villi of the duodenum, jejunum, and ileum and increased lysosomal storage in the submucosa throughout the gastrointestinal tract [24].

Thomas et al. showed that gastrointestinal complications caused or contributed to 5.9 % of deaths in this population in their study of patients with MPS III.

To that end, the increase in GAG levels in patients with MPS disorders may cause inflammation in intestinal cells in the intestine or cause deterioration of iron absorption from intestinal cells. The potential association of hematological abnormalities and MPS manifestations highlights the importance of exploring the role of regular iron studies and hematological tests in at risk MPS patients as undiagnosed anemia has been shown to worsen clinical outcomes.

Importantly, urine GAGs were not raised in older patients with attenuated forms such as MPS I Scheie (Table 1). Age related reference ranges for urine GAGs have been previously derived by our group [25], and confirmed that older patients and those with attenuated forms may have low and normal urine GAGs. It was also reflected by the lack of correlation between urine GAGs and serum iron as shown in Fig. 4.

In this study we observed that the negative correlation between patients' serum iron concentration and urine GAG excretion in MPS III confirms that excessive GAG accumulation may be associated with a limited iron absorption (Suppl Fig. 2). Serum iron concentration does not accurately evaluate the body iron stores. Other clinical tests, such as serum ferritin, transferrin saturation, soluble transferrin receptor, or C-reactive protein, proved to be useful adjunctive tests to assist in the diagnosis of iron deficiency [26]. Ferritin is a sensitive marker of iron deficiency in chronic diseases with no obvious inflammation or infection. Ferritin concentration below 100 μg/L is believed to be an indicator of limited iron storage (personal observations) and this is what has been observed in our study.

All the MPS patients have serum B12 and folate measured routinely (Supplementary Fig. 3). Deficiencies of serum B12 and folate have been observed in these cohort and may be associated with raised MCV, with a positive correlation of 0.27. (Fig. 2).

Pancytopenia was observed in treatment naïve adults affected with MPS III disorders and was associated with bone marrow infiltration. Although, liver function test abnormalities were not apparent, further abdominal imaging was pursued. All these cases had a significant splenomegaly and urine GAGs were 7-to-20-fold upper reference range (Supplementary Fig. 2). These patients also had remarkable serum iron studies abnormalities.

### 4.4 Cardiac valve disease

Cardiac involvement is seen in patients with MPS disorders. This condition, which also affects the patient's life expectancy, is difficult to control. Anemia of various origin developing in the patients may aggravate cardiac outcomes [27].

Intravascular destruction of erythrocytes sufficient to produce hemolytic anemia occurs with incompetent prosthetic heart valves. First reports of increased urinary iron loss eventually producing an iron deficiency come from 1960s and 70s [27,28]. Hemolysis developing in patients with valve disease may contribute to profound anemia and iron deficiency. It has been also observed that in normally functioning prosthetic heart valves, subclinical hemolysis is a common finding. A low incidence of hemolysis has been documented in stented biologic prostheses, and it is absent in stentless aortic valves [29].

Increases in urine GAGs in these patients may be multifactorial. A pause in ERT perioperatively is one of the recognized caused or raised urinary GAG excretion.

### 4.5 Hematopoietic stem cell transplantation

Currently available treatments include hematopoietic stem cell transplantation (HSCT) [30,31], enzyme replacement therapy (ERT), [[32], [33], [34], [35], [36]]. The available therapeutic modalities slow disease progression rather than curing patients, particularly because treatment is often started after organ damage has already occurred [1,3]. Due to the longer life expectancy offered by drug therapy, new complications and issues appear that need to be treated to improve the adult patients' quality of life [37].

Historically, myeloablative (MA) and nonmyeloablative (NMA) therapies have been given to transplant patients. Before 2000, mortality rates associated with HSCT for MPS were reported as high as 27 % [32,38]. Total body irradiation has not been used in MPS IH patients since 2002 because total body conditioning regimens have adversely affected neurodevelopment, growth, hypothyroidism, and cataracts at the extremes. [32]. Radiotherapy in MPS patients may complicate the existing intestinal involvement. The already existing intestinal damage in MPS patients may be further aggravated by radiotherapy, and the long-term aging effect of radiotherapy has not been evaluated in MPS disorders so far.

In contrary, in a mouse model the small intestine has been shown to be less sensitive to radiation than the hematopoietic cells, as a result of its rapid response to and repair of damage [39], so the total body irradiation dose for HSCT didn't result in obvious gastrointestinal complications. As a late effect, however, small intestine senescence persisted in a mouse [40]. It was therefore concluded that results indicated that long-term intestinal abnormalities in digestion and absorption caused by irradiation can remain a concern in adults who underwent HSCT in childhood.

In non-MPS patients, post-transplant enteritis and related anemia can frequently be observed in the acute phase after the procedure [41]. Persistent anemia or iron deficiency anemia are not commonly seen in adults post HSCT (personal observations).

In a population of patients with MPS disorders who underwent HSCT and total body irradiation, Qu et al. have shown that hemolytic anemia was reported in 8.4 % of the patients [42]. Autoimmune hemolytic anemia was documented up to 9 months post HSCT in MPS IIB child [43] and resolved after 14 months of corticosteroid therapy. There was no mention of iron deficiency anemia. In this study, eleven of patients with MPS IH had radiotherapy used as part of the conditioning regimen HSCT, and anemia was observed in seven of them. Urine GAGs were raised in four patients (Suppl Fig. 4).

The cohort of adult patients required repeated courses of enteral iron replacement. Due to lack of clinical and biochemical response, four patients required iron transfusion. Celiac disease and inflammatory disease were excluded before intravenous iron replacement. Noteworthy all four patients were females; two experienced menorrhagia, one had a previous history of major cardiac surgery. One other patient had a history of glomus tumors, which might have contributed to her anemia.

Several therapies are currently in development, including new generation ERT targeting transferrin receptor (TfR) for brain delivery. The receptor is highly expressed on brain endothelial cells and undergoes constitutive ligand-independent endocytosis [44].

The small sample sizes for each subtype of MPS is a limitation of the study. In addition, the study has no controls although the attempts were made to compare the results with the prevalence of iron deficiency anemia in the general population.

The authors are aware that the phenotypic expression of MPS patients can vary from Hurler to Scheie-like forms, creating a further bias in the interpretation of the results. Although, the analysis was separating MPS I subtype respective of the therapy, the phenotype may differ with a further impact on the iron and hematological parameters.

## 5 Conclusions

Iron metabolism in MPS disorder is underexamined. Anemia in these conditions is commonly observed, with iron deficiency anemia being the most prevalent.

Health care professionals need to be aware of hematological alterations in patients with MPS disorders and correlate them with patient symptoms and other clinical laboratory results.

It is important to take them into account in the management of MPS patients, as special approaches may be required for both intestinal health and anemia treatment.

In addition, it is necessary to extend the study of hematological alterations to children with MPS disorders to explore the potential of these abnormalities as a biomarker for diagnosis or treatment follow-up.

## CRediT authorship contribution statement

**Gabija Kaciulyte:** Validation, Resources, Methodology, Formal analysis, Conceptualization, Writing – original draft, Software, Project administration, Investigation, Data curation. **Goknur Yorulmaz:** Software, Investigation, Validation, Methodology. **Reena Sharma:** Investigation, Methodology. **Simon A. Jones:** Data curation, Project administration. **Robert Wynn:** Resources, Data curation, Supervision, Investigation. **Heather Church:** Data curation, Resources. **Karen Tylee:** Resources, Data curation, Software, Methodology. **Muzaffer Bilgin:** Validation, Resources, Investigation, Data curation, Software, Methodology, Formal analysis. **Ana Jovanovic:** Data curation, Writing – review & editing, Resources, Investigation. **Peter Woolfson:** Supervision, Investigation, Validation, Methodology, Data curation. **Karolina M. Stepien:** Writing – review & editing, Validation, Resources, Methodology, Formal analysis, Conceptualization, Writing – original draft, Supervision, Project administration, Investigation, Data curation.

## Institutional review board statement

The project was registered as a service evaluation project in Salford R&I.

## Funding

No funding was available for this project.

## Declaration of competing interest

The authors have no conflict of interest for this study.

## Data Availability

Data will be made available on request.

## Appendix A Supplementary data

Supplementary material Image 1
