# Supplementary material for: Iron metabolism and hematological abnormalities in adult patients affected with mucopolysaccharidoses
Source: Mol Genet Metab Rep. 2025 Jul 16;44:101243. doi: 10.1016/j.ymgmr.2025.101243 (PMC12284490; doi:10.1016/j.ymgmr.2025.101243)

Supplementary Figure 1


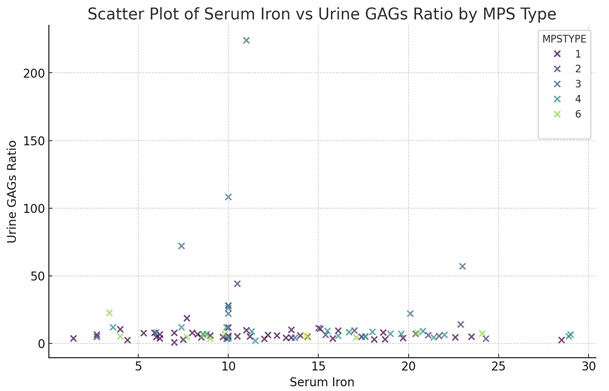


Supplementary Figure 2


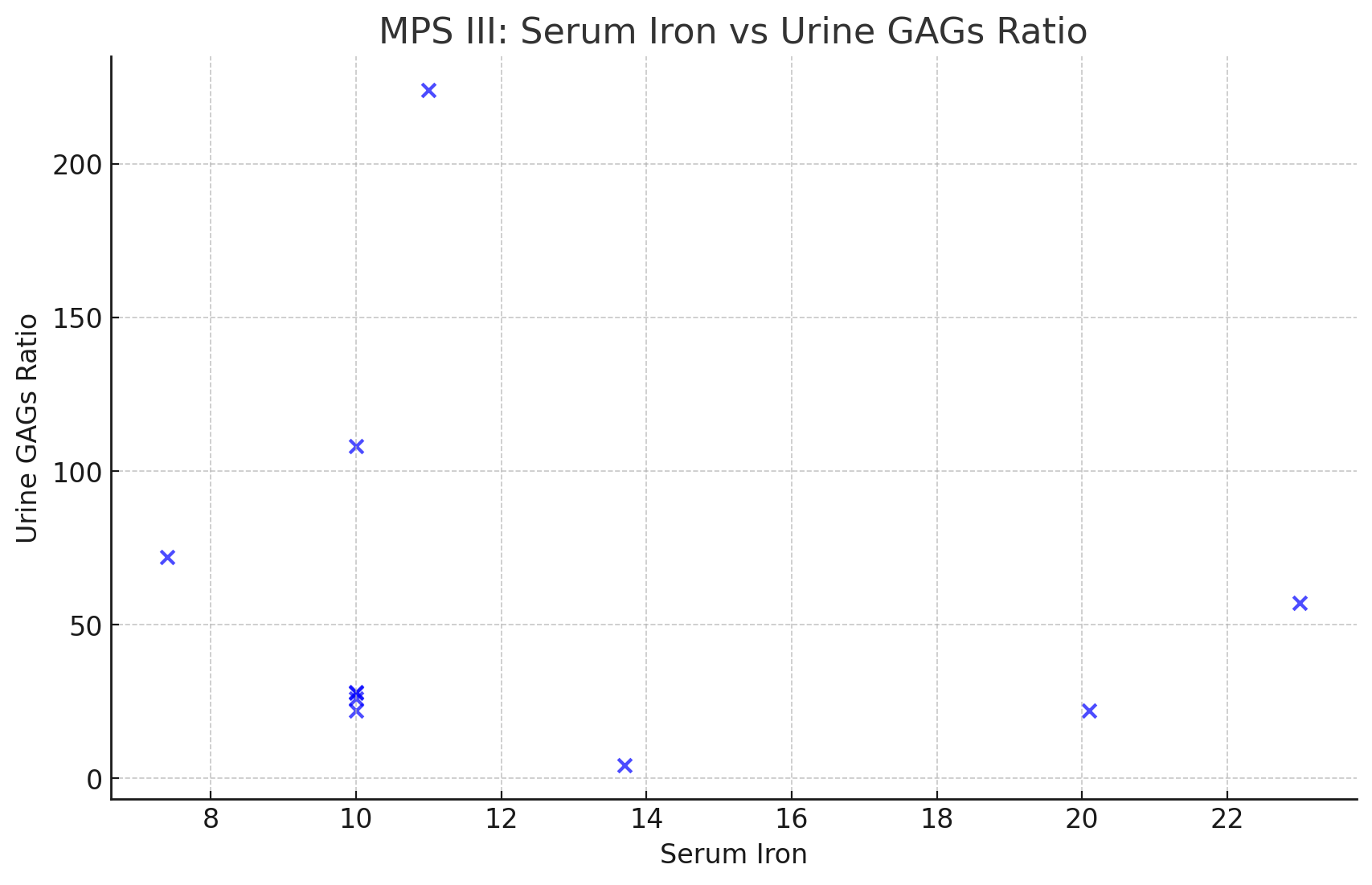


Supplementary Figure 3: Median values of haematological parameters and haematinics


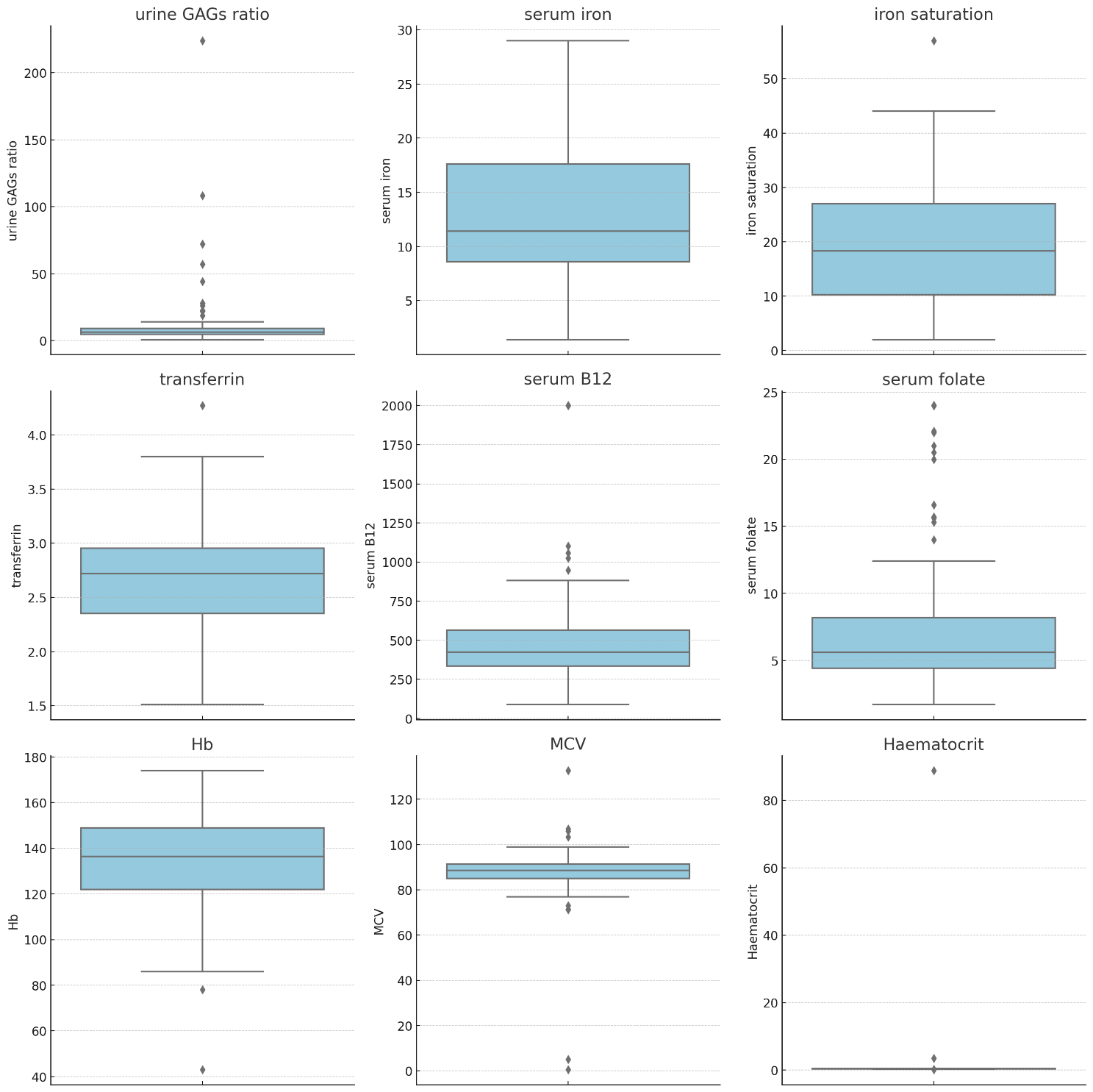

Supplement: Supplementary file 1 — Supplementary material [file mmc1.docx]
